# Production of Infectious Genotype 1b Virus Particles in Cell Culture and Impairment by Replication Enhancing Mutations

**DOI:** 10.1371/journal.ppat.1000475

**Published:** 2009-06-12

**Authors:** Thomas Pietschmann, Margarita Zayas, Philip Meuleman, Gang Long, Nicole Appel, George Koutsoudakis, Stephanie Kallis, Geert Leroux-Roels, Volker Lohmann, Ralf Bartenschlager

**Affiliations:** 1 Department of Molecular Virology, University of Heidelberg, Heidelberg, Germany; 2 Center for Vaccinology, Ghent University and Hospital, Ghent, Belgium; The Rockefeller University, United States of America

## Abstract

With the advent of subgenomic hepatitis C virus (HCV) replicons, studies of the intracellular steps of the viral replication cycle became possible. These RNAs are capable of self-amplification in cultured human hepatoma cells, but save for the genotype 2a isolate JFH-1, efficient replication of these HCV RNAs requires replication enhancing mutations (REMs), previously also called cell culture adaptive mutations. These mutations cluster primarily in the central region of non-structural protein 5A (NS5A), but may also reside in the NS3 helicase domain or at a distinct position in NS4B. Most efficient replication has been achieved by combining REMs residing in NS3 with distinct REMs located in NS4B or NS5A. However, in spite of efficient replication of HCV genomes containing such mutations, they do not support production of infectious virus particles. By using the genotype 1b isolate Con1, in this study we show that REMs interfere with HCV assembly. Strongest impairment of virus formation was found with REMs located in the NS3 helicase (E1202G and T1280I) as well as NS5A (S2204R), whereas a highly adaptive REM in NS4B still allowed virus production although relative levels of core release were also reduced. We also show that cells transfected with the Con1 wild type genome or the genome containing the REM in NS4B release HCV particles that are infectious both in cell culture and *in vivo*. Our data provide an explanation for the *in vitro* and *in vivo* attenuation of cell culture adapted HCV genomes and may open new avenues for the development of fully competent culture systems covering the therapeutically most relevant HCV genotypes.

## Author Summary

The hepatitis C virus (HCV) is a major cause of acute and chronic liver disease. Unusual for a positive strand RNA virus, HCV has the high propensity to establish persistent infection, which increases the risk for liver cirrhosis and hepatocellular carcinoma. No selective therapy is available thus far and its development has been hampered by the lack of adequate cell culture systems. With the advent of subgenomic replicons, i.e. RNAs containing only the viral replicase genes and that self-amplify in the human liver cell line Huh-7, this hurdle has been overcome to some extent. However, save for a single genotype 2a isolate, efficient replication of all HCV isolates described thus far requires replication enhancing mutations (REMs), but genomes with REMs do not support production of infectious virus particles. In this study we show that except for one mutation in non-structural protein 4B, REMs interfere with the assembly of infectious virus particles, whereas an unaltered HCV genome supports production of cell culture–derived virus that is infectious *in vitro* and *in vivo*. Our observations provide an explanation for the attenuation of cell culture adapted HCV genomes and open new perspectives for the development of culture systems for difficult to treat HCV genotypes.

## Introduction

**Figure 1:**
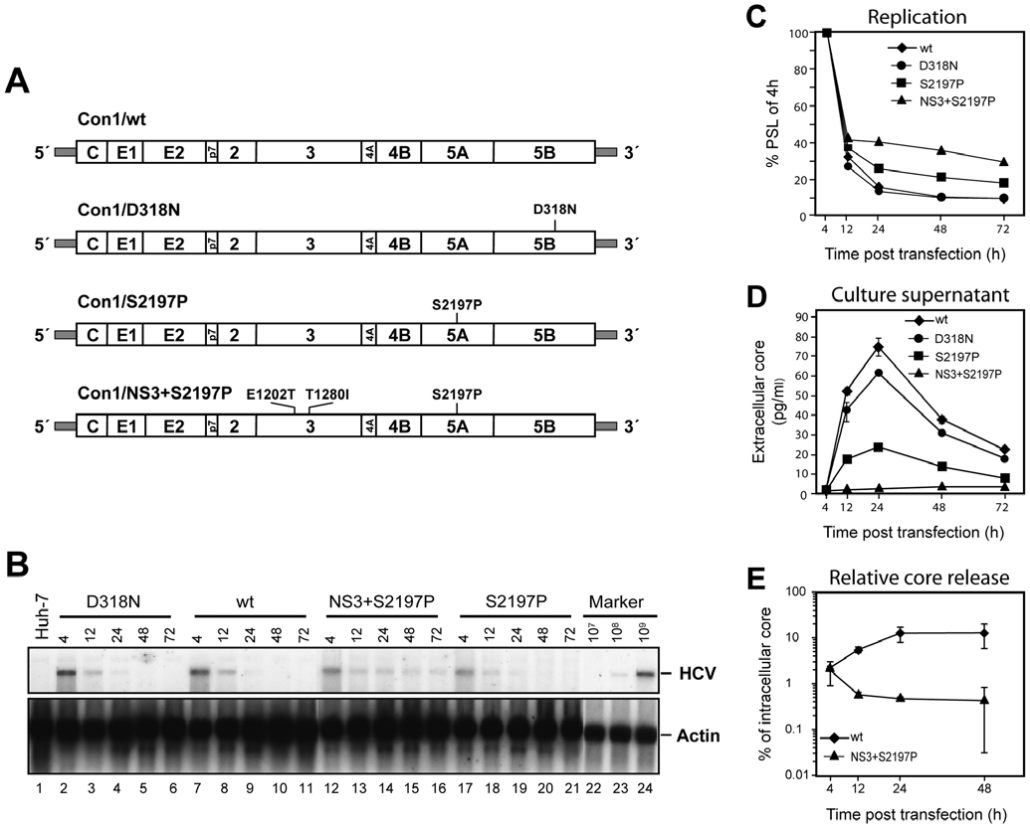
Transient replication of HCV Con1-derived constructs in Huh-7 cells and release of core protein from transfected cells. (A) Schematic representation of transfected RNA genomes. The polyprotein is indicated by an open box, the individual functional proteins are separated by vertical lines. Non-translated regions are depicted as shaded bars, REMs and the position of the mutation destroying the active site of the NS5B RdRp (D318N) are specified above the respective positions in the coding region. (B) Transient RNA replication of full length Con1-derived genomes. Ten µg of *in vitro* transcribed RNA of the constructs specified in the top were transfected into Huh-7 cells that were harvested at given time points. Total cellular RNA was prepared and HCV RNA and beta-actin RNA were detected by Northern hybridization. (C) The amount of HCV RNA was determined by phospho imaging and is expressed relative to the input determined 4 h post transfection. Values were normalized for equal RNA loading as determined with the beta-actin specific signal. (D) Time course of accumulation of HCV core in cell culture supernatant of transfected Huh7 cells. Cells were transfected and seeded as described in (A). At given time points, culture medium was harvested, filtered through 0.45 µm pore-size filters, and analysed for core protein by ELISA. Duplicate measurements, mean value of duplicates and standard errors of the means are given. (E) Efficiency of core release from cells transfected with Con1/wt or the adapted genome Con1/NS3+S2197P [27]. Amounts of core protein accumulated intracellularly or in cell culture medium were determined by ELISA and used to calculate the percentage of intracellular core protein released into the supernatant of transfected cells for each given time point. Mean values of two independent electroporations including standard errors of the means are shown.

HCV is a positive strand RNA virus which belongs to the family *Flaviviridae* [1]. Its genome of about 9.6 kb is composed of the 5′non-translated region (NTR), an open reading frame encoding a large polyprotein, and the 3′NTR [2] (Fig. 1A). In the N-terminal region, the polyprotein is processed by cellular proteases to yield the structural proteins Core (C), envelope proteins 1 and 2 (E1, E2), and p7. Cleavage of the non-structural (NS) proteins is accomplished by NS2 at the NS2/3 site and by the NS3 protease at all remaining sites [2]. NS4B induces cellular membrane alterations thought to provide a scaffold for the viral replication machinery [3],[4]. NS5B is the viral RNA-dependent RNA polymerase whereas NS5A is an RNA binding phosphoprotein involved in RNA replication and virus assembly [5]–[8]. Two NS5A phospho variants have been described assumed to correspond to a basal and a hyper phosphorylated form (p56 and p58, respectively) [9]. Phosphorylation of NS5A appears to be mediated by casein kinase I and II [7],[10]. Interestingly, interference with NS5A hyperphosphorylation by inhibitors of casein kinase I such as H479 enhances viral RNA replication by more than 10-fold arguing that this modification is of disadvantage for high level replication [11].

About 170 million people are chronically infected with HCV. At present, neither a selective antiviral therapy nor a vaccine is available, and only a fraction of patients treated with a combination of polyethylene glycol (PEG)-conjugated interferon alpha (IFN-α) and ribavirin can be cured [12]. Thus, there is an urgent need for more effective antiviral treatment that also has fewer side effects. The development of such therapeutics and vaccines has long been hampered by the notoriously poor replication of HCV in cultured cells. The advent of subgenomic replicons that were originally derived from the genotype 1b isolate Con1 and that amplify efficiently in the human hepatoma cell line Huh-7 has in part overcome this limitation [13]. However, for Con1 mutations within the viral NS proteins are required to increase RNA replication to levels sufficient for experimental analyses [14]–[17]. These mutations have originally been designated ‘cell culture adaptive mutations’, but should be renamed as ‘replication enhancing mutations’ (REMs) in order to discriminate them from cell culture adaptive mutations that increase virus titers without affecting replication [18]–[23]. The latter will therefore be designated ‘titer enhancing mutations’ (TEMs) throughout this report. REMs have been identified with all replicons derived from genotype 1 HCV isolates (reviewed in [24]). These mutations cluster primarily in the center of NS5A and at distinct positions in NS3 and NS4B. Certain combinations of mutations enhance RNA replication cooperatively, the exact mode of action, however, remains elusive.

The superior RNA replication capacity accomplished by adapted NS-proteins allowed the generation of efficiently replicating full length HCV genomes [25],[26]. However, in spite of high level replication, these genomes do not or only very poorly support the production of detectable levels of infectious HCV particles [25],[26]. Earlier we demonstrated that the presence of certain REMs interferes with infectivity *in vivo* [27]. A Con1 genome with a single mutation in NS5A (S2197P) was severely attenuated in chimpanzees inoculated intrahepatically with *in vitro* transcripts of this genome. A triple mutant carrying two mutations in NS3 (E1202G, T1280I) and the aforementioned substitution in NS5A was unable to establish a productive infection implying that REMs interfere with infectivity *in vivo*. In line with this assumption, efficient production of infectious HCV in cell culture so far has only been achieved with a genotype 2a HCV isolate (designated JFH-1) that replicates to very high levels without requiring REMs [28],[29].

Employing transient replication and virus release assays in this study we demonstrate that REMs interfere with the production of infectious HCV particles. We show that at least in case of the Con1 isolate, mutations especially in the NS3 helicase, but also in NS5A and NS5B lead to strong impairment of virus production whereas Huh-7 cells transfected with the wild type genome or a genome containing one REM in NS4B release substantial amounts of HCV that is infectious in cell culture and *in vivo*.

## Results

### Interference of REMs with virus production

Given the infectivity of the wild type Con1 HCV isolate *in vivo* on one hand and its poor replicative capacity in transfected Huh-7 cells *in vitro* on the other hand, we first investigated whether this genome is capable of producing virus particles in cell culture. Four constructs were used for this experiment: Con1/wild type (wt), a replication incompetent variant thereof with a mutation that destroys the active site of the polymerase (Con1/D318N), a weakly adapted Con1 genome containing a REM in NS5A (Con1/S2197P) and a highly adapted Con1 genome containing two REMs in NS3 (E1202T, T1280I) and one in NS5A (S2197P) and designated Con1/NS3+S2197P (Fig. 1A). We have shown earlier that the combination of these 3 REMs enhances RNA replication in a cooperative manner [15] *In vitro* transcripts were transfected into Huh-7 cells and RNA replication was quantified by Northern blotting and phospho imaging (Fig. 1B and C, respectively), whereas virus production was measured by determining the release of core protein into the supernatant of transfected cells (Fig. 1D). As expected Con1/wt replicated poorly in Huh-7 cells, displaying no clear difference to the replication incompetent mutant Con1/D318N. In contrast, elevated RNA levels were observed for the weakly and highly adapted genomes (Con1/S2197P and Con1/NS3+S2197P, respectively). Analysis of extracellular core protein levels revealed that highest amounts of core were transiently released into the supernatant of cells transfected with Con1/wt (Fig. 1D). Core protein was first detectable at 12 h post transfection, increased to peak levels at 24 h, and declined rapidly thereafter. This kinetic correlated well with intracellular RNA levels with highest amounts of Con1/wt input RNA detected right after transfection followed by a rapid decline due to the poor replication of this genome (Fig. 1D). Interestingly, the same kinetic of core release was found with the replication inactive Con1/D318N mutant demonstrating that at least in this setting core release does not require RNA replication. Most importantly, the Con1 variants containing the REMs displayed clearly impaired core release. This defect was most prominent for the triple mutant Con1/NS3+S2197P which despite highest intracellular RNA and core quantities did not release core protein to detectable levels. Coherently, Con1/S2197P harbouring a single REM in NS5A exhibited impaired release of core relative to Con1/wt, although due to the adapted phenotype, intracellular RNA levels were elevated. In contrast, about 15% of intracellular core protein was released from cells transfected with Con1/wt (Fig. 1E). The inverse correlation between increase of RNA replication by the REMs in NS3 and NS5A and the impaired core release suggested that such mutations interfere with virion production.

**Figure 2:**
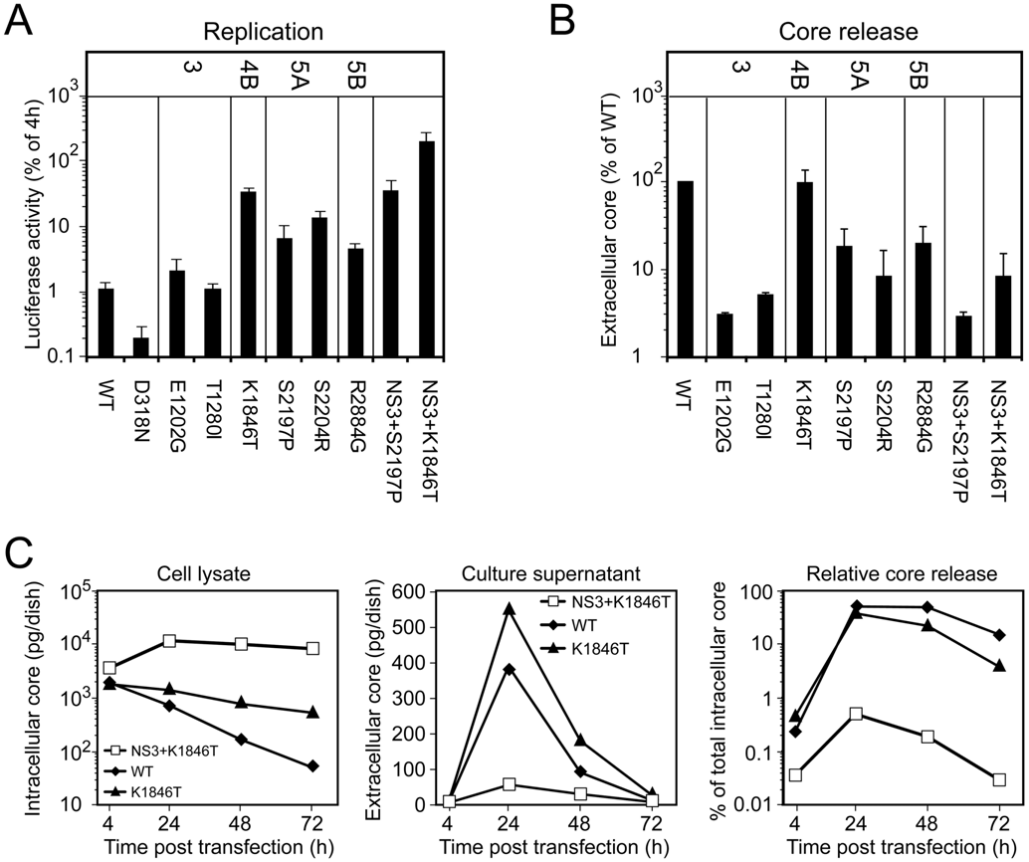
Impact of REMs on HCV particle release. (A) Replication of subgenomic luciferase replicons in transfected Huh-7 cells. The replication deficient replicon D318N served as negative control. Values refer to luciferase activities determined 48 h post transfection of Huh-7 cells after normalization for transfection efficiency determined 4 h post transfection. Data are taken from reference [17]. (B) Huh-7 cells were transfected with the Con1/wt genome or Con1 genomes carrying REMs specified in the bottom of the graph. The amount of core protein released into the supernatant 24 h after transfection was determined by using core ELISA and is expressed relative to the quantity of core measured for Con1/wt. Mean values of between 2 and 11 independent repetitions (depending on the construct) including the standard deviation are given. (C) Impact of REMs in NS4B and NS3 on RNA replication as determined by intracellular accumulation of core protein (left panel). The kinetic of release of core protein into the supernatant of Huh-7 cells after transfection with the Con1/wt or the Con1/K1846T or the Con1/NS3+K1846T genome is shown in the middle panel. The right panel shows the percentage of intracellular core that is released into the culture supernatant of Huh-7 cells at various time points after transfection with each of the 3 genomes. A representative example of two independent repetitions is shown.

To more quantitatively determine the correlation between the extent of replication enhancement by a given REM and the impairment of virus production we generated a panel of Con1-derived subgenomic reporter replicons and full length genomes into which single REMs were inserted. For this purpose we selected several representative REMs residing in NS3 (E1202G, T1280I), or NS4B (K1846T), or NS5A (S2197P, S2204R), or NS5B (R2884G) that increase replication of subgenomic HCV replicons to various extents (Fig. 2A) [17]. In addition, we analyzed two different combinations of mutations in which the two REMs in NS3 were combined either with the REM in NS4B (construct Con1/NS3+K1846T) or NS5A (construct NS3+S2197P). As shown in Fig. 2A amongst the single mutations, the REM located in NS4B (K1846T) enhanced RNA replication most whereas the two mutations residing in NS3 had least effects [15],[17]. As described earlier, when these two REMs in NS3 were combined either with the mutation in NS4B or the S2197P substitution in NS5A, RNA replication was enhanced cooperatively (replicons NS3+K1846T and NS3+S2197P, respectively) [17].

To determine the impact of these mutations on core release they were inserted into the parental Con1 full length genome. Subsequently, mutants were transfected into Huh-7 cells and core release was determined by ELISA. The results in Fig. 2B show that strongest inhibition of virus production (assembly or release) was exerted by either of the two REMs in NS3 that enhanced RNA replication only to a minor extent. Profound impairment of core release was mediated by the REMs residing in NS5A or NS5B whereas the REM in NS4B still allowed core release to a level comparable with the wild type. However, when this mutation or the S2197P REM in NS5A was combined with the two mutations in NS3 core release was potently blocked.

Given the rather efficient core release obtained with the Con1/K1846T genome, we performed a more detailed quantitative analysis by measuring the accumulation of intra- and extracellular core protein amounts at various time points post transfection (Fig. 2C). In agreement with the elevated replication level, the amounts of intracellular core protein were consistently higher in cells transfected with Con1/K1846T as compared to Con1/wt transfected cells, but substantially lower compared to Con1/NS3+K1846T (Fig. 2C, left panel). Likewise, amounts of core protein released into the supernatant of transfected cells were elevated in case of Con1/K1846T transfected cells (middle panel). However, when correlating the amount of released core protein to the total amount of core protein expressed we found that also the NS4B mutation reduced core protein release, especially at later time points post transfection (48 and 72 h; Fig. 2C, right panel). In summary, our data suggest that REMs, at least those examined here, have a negative impact on core release, but the extent of this interference does not correlate with the extent of RNA replication enhancement.

### Role of envelope glycoproteins for Con1/wt particle production

**Figure 3:**
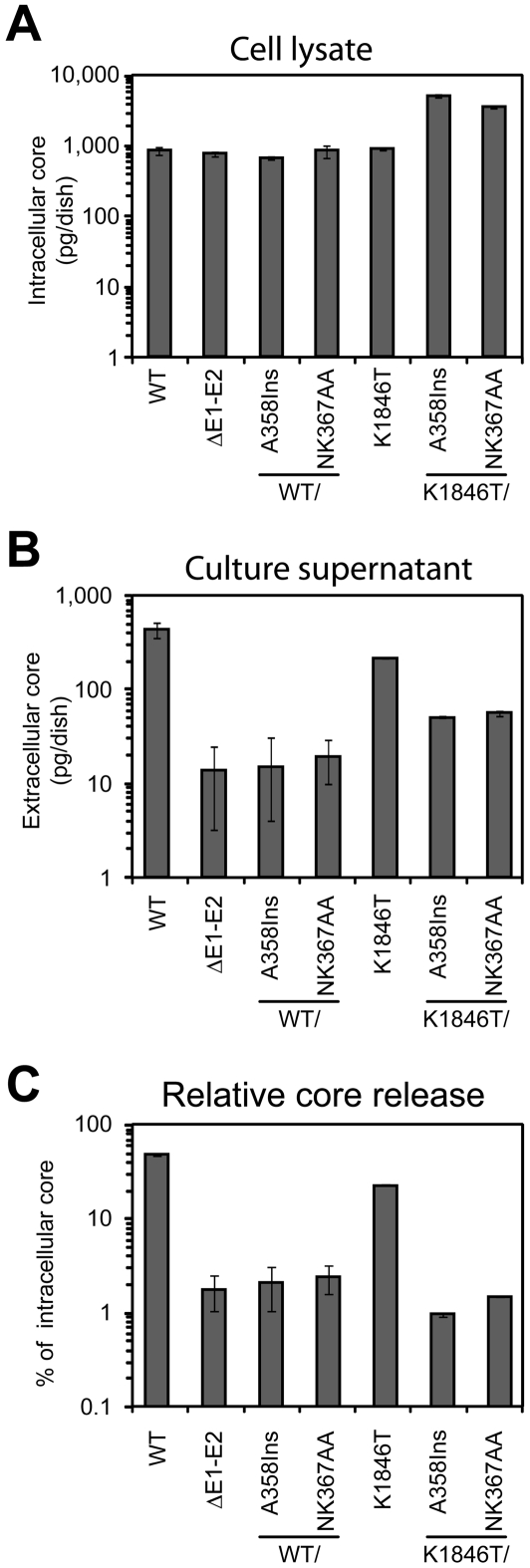
Release of HCV core into the supernatant of transfected cells depends on the expression of functional glycoproteins. Ten µg of *in vitro* transcribed RNA of the constructs specified below each bar were transfected into Huh-7 cells and 24 h later culture medium was harvested and filtered, whereas cells were lysed with 1% Triton X-100 in PBS. The total amount of HCV core in the cell lysate (A) and culture supernatant (B) was determined by ELISA. (C) Relative core release expressed as the fraction (in %) of total intracellular core protein that is released into the culture fluid. Mean values of two independent electroporations including the standard error of the means are given.

To confirm that core protein released into supernatants of cells transfected with Con1-derived constructs represents virus particles, we studied the requirements for core release by using reverse genetics. Several variants of the Con1 genome were generated: Mutant ΔE1-E2 comprising a large in frame deletion that encompasses most of the E1 and E2 coding sequence; mutant wt/A358Ins and K1846T/A358Ins in which an alanine codon was inserted into the E1 coding region at amino acid residue 358; mutants wt/NK367AA and K1846T/NK367AA in which two amino acid residues within the transmembrane domain of E1 were replaced by alanine residues. The A358Ins and NK367AA mutations were previously shown to disturb E1/E2 heterodimerization thereby blocking formation of functional glycoprotein complexes while the deletion of E1-E2 is known to abrogate release of infectious JFH1 particles [28],[30],[31]. Twenty four hours after transfection, total amount of core protein present in cell lysate and medium was determined by core-specific ELISA. Con1 wild type genomes expressed comparable amounts of core in the cell lysate arguing for comparable transfection efficiency (Fig. 3A). Owing to higher replication, core amounts in cells transfected with the K1846T-constructs were higher. In case of the wild type up to about 45% of intracellular core protein was released, whereas in case of the K1846 mutant this value was reduced to about 20% (Fig. 3C). Most importantly, core release was reduced to background levels (as determined with the ΔE1/E2 mutant) whenever the mutations in the envelope coding sequence were introduced and this effect was found both with the wt and the K1846T genome (Fig. 3C). This result indicates that core release observed with these two genomes is a specific process that requires functional envelope glycoproteins.

**Figure 4:**
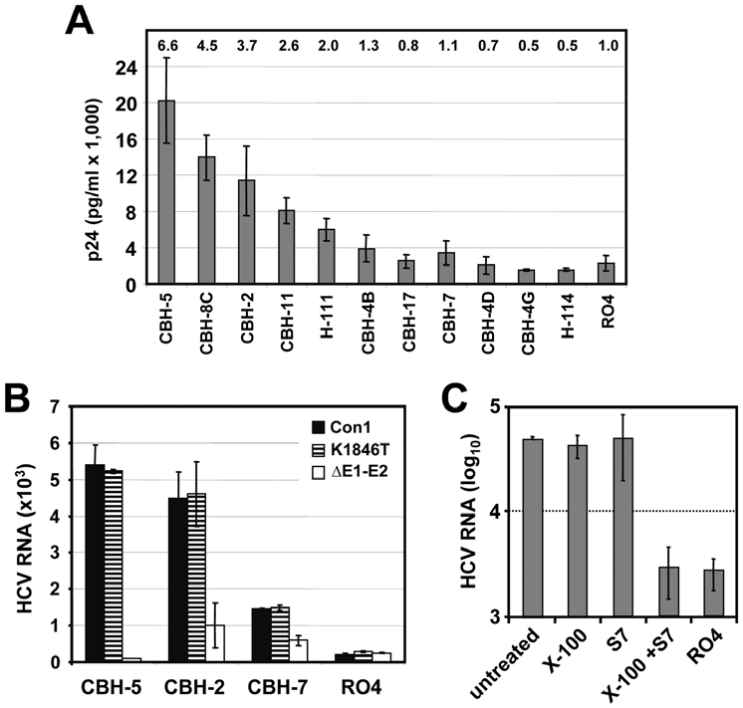
Capture of Con1 particles by E2-specific antibodies and characterization of captured particles. (A) Identification of monoclonal antibodies reacting with Con1 envelope glycoproteins. HCVpp carrying Con1 envelope glycoproteins were incubated with immobilized antibodies specified in the bottom and captured particles were quantitated by using p24-specific ELISA. Numbers above each bar refer to fold increase above background as determined by capture assay with an irrelavant antibody (RO4). Mean values of three experiments and the standard deviations are given. (B) Capture of HCV particles from supernatants of Huh-7 cells transfected with Con1/wt or Con1/K1846T or the Con1 mutant lacking most of the envelope glycoprotein coding region (ΔE1–E2). Bound particles were quantified by using TaqMan qRT-PCR. Mean values of triplicate measurements including the standard error of the means are given. (C) Characterization of captured Con1/wt particles by nuclease treatment. Concentrated culture supernatant of Con1/wt transfected cells was used for capture with control antibody R04 (right bar) or CBH-5 (all other bars). Immune complexes obtained with the latter were split into 4 aliquots that were left untreated or treated with 0.5% Triton X-100, or nuclease S7, or 0.5% Triton X-100 and S7 nuclease. RNA was extracted and quantified by TaqMan qRT-PCR.

If authentic virus particles were produced from Con1/wt or Con1/K1846T transfected cells, the core protein shell which harbours the viral RNA should be surrounded by a lipid membrane containing the viral envelope glycoproteins. Consequently, core protein and HCV RNA should be captured by antibodies directed against E1 or E2. To identify HCV-envelope specific antibodies to be used for Con1 virus capture assay we first screened a panel of human monoclonal antibodies recognizing HCV E2 or E1 proteins for their capacity to bind to HCV pseudo particles (HCVpp) generated in 293T cells by using an HIV-based vector and a HCV Con1 E1–E2 expression construct. The isotype-matched RO4 antibody which is directed against p64 of Cytomegalovirus (CMV) served as negative control. Upon incubation with monoclonal antibodies the amount of captured HCVpp was determined by using HIV p24-specific ELISA. As shown in Fig. 4A, highest yields were obtained with the human monoclonal antibodies CBH-5, CBH-8C and CBH-2 [32]. For subsequent capture assays we used CBH-5 and CBH-2, two efficient capture antibodies as well as CBH-7 that captured only low amounts of Con1-derived HCVpp and thus served as sensitivity control. The RO4 antibody was used as negative control. Concentrated cell culture supernatants derived from Con1/wt, Con1/K1846T and Con1/ΔE1/E2 transfected Huh-7 cells were incubated with antibody coupled beads and the quantity of captured HCV RNA was determined by qRT-PCR after extensive washing with PBS (Fig. 4B). Although the overall capture efficiency was only about 5%, particles present in supernatant of Con1/wt and Con1/K1846T transfected cells could specifically be captured with all 3 CBH-antibodies. Capture efficiency correlated well with the one obtained with HCVpp. Only background amounts of RNA were captured from the supernatant with the non-specific antibody (RO4) or from supernatant of Con1/ΔE1-E2 transfected cells demonstrating specificity of this capture assay.

### Biophysial characterization of Con1-derived particles

Biophysical properties of captured particles were further characterized by treatment with S7 micrococcal nuclease under various conditions. Assuming that intact particles protect the viral genome, we treated captured complexes with nuclease without or with prior treatment with the detergent Triton X-100 (Fig. 4C). We found that the viral genome in captured complexes was resistant to nuclease treatment consistent with the protection of the RNA by an intact virus particle. Removal of lipids however, by detergent treatment rendered the viral genome fully nuclease sensitive and RNA levels after Triton X-100 and S7 treatment were at the background as determined with the capture with the R04 control antibody. Addition, of protease to the detergent-treated particles did not increase nuclease sensitivity (not shown). These results suggested that HCV nucleocapsids –if they are formed at all- might be unstable after removal of the envelope and/or the lipoprotein shell.

**Figure 5:**
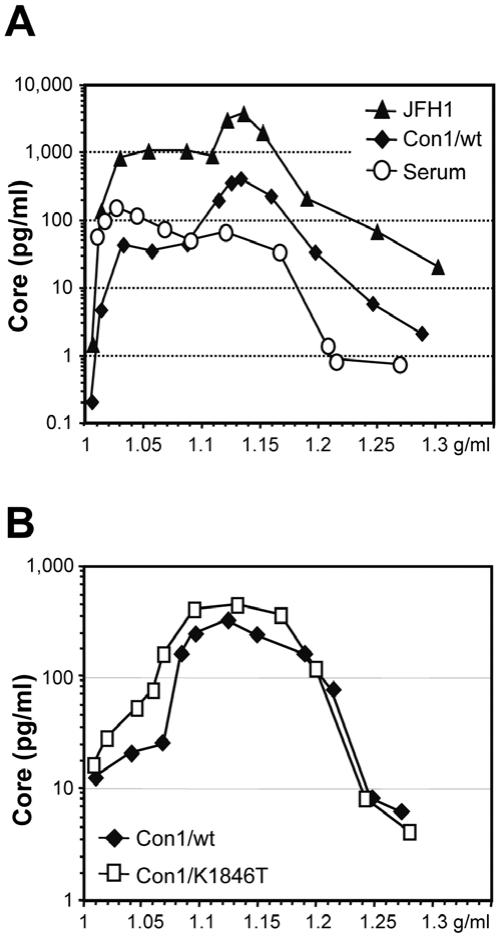
Density profiles of HCV particles derived from Con1/wt, Con1/K1846T, JFH-1 and patient serum. (A) Approximately 22 ml of filtered cell culture fluid harvested 24 h after transfection of Huh-7 cells with Con1/wt RNA were concentrated via ultracentrifugation over a 60% iodixanol cushion. In case of JFH-1 approximately 26 ml supernatant harvested 96 h post transfection and concentrated in the same manner were used. The concentrates were overlaid with a linear iodixanol gradient (0%–60%) and spun for 20 h at 110,000 g at 4°C. In a similar experiment, 0.5 ml of high titer patient serum was resolved in an iodixanol density gradient. Twelve fractions à 1ml were harvested from the top, and the amount of HCV core protein contained in each fraction was determined by Trak C ELISA, respectively. HCV core content per fraction is plotted against the density of the respective fraction. (B) Result of an analogous experiment but using culture supernatant of Huh-7 cells after transfection with Con1/wt or Con1/K1846T.

Densities of Con1-derived particles were determined by using density gradient centrifugation in comparison to virus particles produced from JFH-1 transfected Huh-7 cells and virus particles contained in a high titer patient serum. Gradient fractions were harvested from the top and core protein amounts contained in each fraction were determined by ELISA. We did not use qRT-PCR because of residual amounts of *in vitro* transcripts and plasmid DNA in virus preparations generated by RNA transfection precluding unambiguous measurements of particle associated HCV RNA. In the first set of experiments we compared particles derived from Con1/wt, JFH-1 and patient serum (Fig. 5A). Most particles present in patient serum had a density of about 1.04 g/ml probably representing viruses associated with very low density and low density lipoproteins [33]–[36]. The minor peak with a density of ca. 1.12 g/ml may correspond to virus that is less complexed with lipoproteins. Interestingly, cell culture-derived Con1/wt and JFH-1 particles exhibited an analogous density profile but with very different ratios when compared to the patient-derived particles. Most particles had a density of 1.14 g/ml, whereas only a minor and broad peak was found at very low density in the range of 1.03 to 1.08 g/ml. In this respect, density profiles of Con1/wt and JFH-1 derived virus particles were indistinguishable. Likewise, density profiles of Con1/wt and Con1/K1846T derived particles were also very similar (Fig. 5B) arguing that the genome with this REM in NS4B is capable of producing virus particles, too.

In summary, the similarity of buoyant densities of cell culture-derived Con1/wt, Con1/K1846T and patient-derived particles supported the notion that authentic HC virions were released from Huh-7 cells transfected with these HCV genomes. The different relative amounts of particles at very low and high densities may reflect differences of lipoprotein ‘imprinting’ of the particles by the host cell (Huh-7 cells versus fully differentiated human hepatocytes) in agreement with the reported defect of Huh-7 cells to produce vLDL [37].

### Infectivity of Con1/wt particles in cell culture

**Figure 6:**
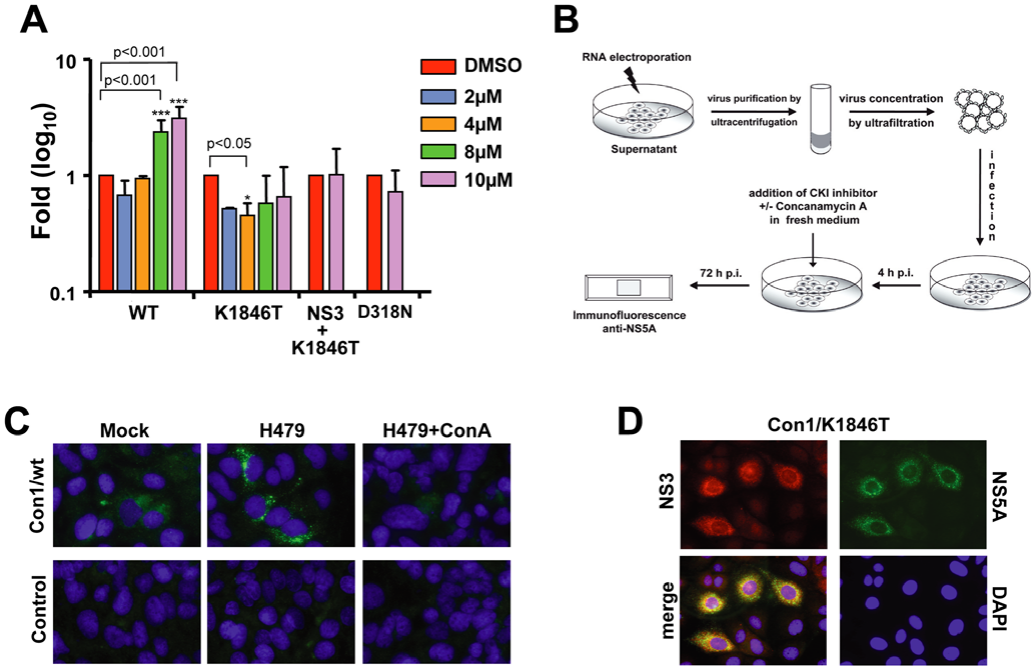
*In vitro* infectivity of Con1/wt particles released from transfected Huh7.5 cells. (A) Enhancement of HCV RNA replication by kinase inhibitor H479. Subgenomic Con1 luciferase replicons were transfected into Huh7.5 cells that were seeded into medium containing H479 at concentrations specified in the right. Cell lysates were prepared at 4 h and 48 h after transfection and luciferase activities were determined. The replication defective replicon Con1/D318N served as negative control. Cells treated with DMSO only were used as reference. For each construct, values were normalized to the luciferase activity of the respective DMSO control in order to determine the fold induction or reduction of replication. Data (mean±S.D.; n = 3) were analyzed using two-way ANOVA test. (B) Experimental approach used to detect *in vitro* infectivity of Con1 virus. (C) Immunofluorescence analysis of Huh7.5 cells 72 h after inoculation with supernatant from cells transfected with the Con1/wt genome (upper panels) or mock transfected cells (lower panels). Cells were treated either with DMSO only (Mock; left panels), or with H479 (middle panels) or with H479 and ConcanamycinA (right panels) as specified in panel (B). Cells were fixed 72 h after inoculation and NS5A was detected by immunofluorescence microscopy. (D) Detection of NS3 and NS5A expression in Huh7.5 cells inoculated with cell-free concentrated supernatant containing Con1/K1846T particles. Cells were fixed 48 h after inoculation and processed for indirect immunofluorescence. Nuclei were counterstained with DAPI.

Attempts to directly demonstrate infectivity of Con1/wt or Con1/K1846T derived particles in cell culture were complicated by the low replicative capacity of both genomes. However, recently Neddermann and colleagues demonstrated that inhibition of casein kinase I that appears to be responsible for hyperphosphorylation of NS5A, with compound H479 results in substantial enhancement of RNA replication of a non-adapted genome whereas replication of a genome containing a REM in NS5A was blocked [10],[11],[38]. Assuming that enhancing replication of Con1/wt and possibly also Con1/K1846T with H479 would facilitate detection of viral proteins in infected cells, we first established the optimal concentration of this kinase inhibitor required to stimulate replication of Con1/wt and Con1/K1846T in Huh7.5 cells. In agreement with the report by Neddermann and colleagues [11] we observed an about 5-fold increase of replication of Con1/wt at 48 h post transfection when Huh7.5 cells were treated with 10 µM of H479 whereas replication of Con1/K1846T was not enhanced (Fig. 6A). Nevertheless, even under optimal conditions replication was about 10–100-fold below the level achieved with the highly cell culture adapted replicon Con1/NS3+K1846T (data not shown).

Having established the optimal conditions for H479-mediated enhancement of Con1/wt replication, we exploited this protocol to determine infectivity of HCV particles in tissue culture (Fig. 6B). Huh7.5 cells, which are more permissive for HCV infection than Huh7-Lunet cells [39] were inoculated with about 200 Con1/wt or Con1/K1846T particles per cell (calculated according to the amount of core protein and assuming 200 core protein molecules per particle) or the analogous volume of concentrated supernatant of mock-transfected cells that was prepared in parallel. Cells inoculated with Con1/wt virus or mock-supernatant were treated with H479 whereas Con1/K1846T inoculated cells were left untreated. Three days after inoculation, cells were fixed and the replicase component NS5A was detected by immunofluorescence. As shown in Fig. 6C, under all conditions no NS5A-specific signal could be detected in cells inoculated with the control-supernatant. In contrast, a low but specific signal was found in Con1/wt inoculated cells and this signal was enhanced in cells that had been treated with the kinase inhibitor H479. Likewise, NS3 and NS5A expression was detected in a few cells inoculated with Con1/K1846T containing supernatant (Fig. 6D). Infection appeared to be a specific process because no signal was detected in Con1/wt inoculated cells that had been treated with Concanamycin A, which is an inhibitor of endosomal acidification and that was shown to block infection of Huh-7 cells with JFH-1 derived virus [40] (Fig. 6C). Infection was also not detected upon infection of Huh7-Lunet cells that express low amounts of CD81 (not shown). These results suggested that cell culture produced Con1/wt or Con1/K1846T particles are infectious *in vitro*. However, quantitative analyses could not be performed due to the low replication of these genomes.

### *In vivo* infectivity of Con1/wt and Con1/K1846T particles produced in cell culture

To firmly demonstrate infectivity of cell culture-produced Con1/wt and Con1/K1846T particles, we performed *in vivo* infection experiments. UPA+/+-SCID mice that had been xenografted with primary human hepatocytes, were inoculated with equally concentrated culture supernatants from Huh7 cells that had been transfected with Con1/wt or Con1/K1846T. As control we used supernatants from cells transfected with Con1/NS3+K1846T, which supports core release to only very low level. Final concentrations of HCV RNA in the purified and concentrated stocks were about 2×10^9^ RNA copies (IU) per ml for all 3 preparations. Based on core-ELISA measurements this corresponded to 5.4×10^3^ pg per ml of Con1/wt, 2×10^3^ pg/ml of Con1/K1846T and 2.3×10^2^ pg per ml core protein of Con1/NS3+K1846T, respectively. Assuming that one HCV particle contains about 200 copies of core protein (an estimate that is derived from hepatitis B virus, which has a similar particle size [41]), the (theoretical) infection dose per animal (100 µl inoculum) was about 7.7×10^7^ particles in case of Con1/wt, 2.9×10^7^ particles in case of Con1/K1846T and about 3×10^6^ particles in case of Con1/NS3+K1846T, respectively. The comparable amounts of RNA detected in all 3 preparations argues that in case of the triple mutant RNA-containing replication complexes that contain no or very low amounts of core protein were released [25].

**Figure 7:**
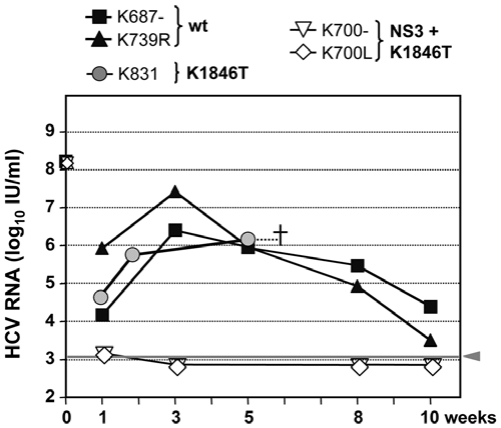
*In vivo* infectivity of Con1/wt, Con1/K1846T and Con1/NS3+K1846T genomes in uPA-SCID mice. Huh7-Lunet cells were transfected with either of these constructs, supernatants were collected 12 and 24 h post transfection, pooled for each construct and used for virus purification and concentration as described in Materials and Methods. Two mice were each inoculated with 2×10^8^ IU HCV RNA per mouse and construct (100 µl inoculum size) and viral RNA loads in sera were determined at the indicated time points after inoculation by qRT-PCR. In case of Con1/K1846T inoculated mice, one died at week 2 (not shown) and the second shortly after week 6. While sera of Con1/wt and Con1/K1846T inoculated mice contained high viral loads already in the first blood sample, Con1/NS3+K1846T-inoculated mice remained HCV RNA negative throughout the 10 weeks observation period.

For each construct, two mice were inoculated with 100 µl of the concentrated stock and virus titers in serum were determined by qRT-PCR (Fig. 7). Unfortunately, one mouse inoculated with Con1/K1846T died spontaneously already at week 2 while the second mouse died at week 6, presumably a follow-up reaction of serum withdrawal. The time course of infection shown in Fig. 7 demonstrates that mice inoculated with Con1/wt or Con1/K1846T particles were readily infected and remained viremic throughout the observation period. In case of the wild type, peak viremia was observed at week 3 post inoculation and steadily declined thereafter, most likely due to a decreased survival of the engrafted human hepatocytes. In contrast, mice inoculated with supernatants from Con1/NS3+K1846T transfected cells remained negative and viral RNA was not detected in any of the serum samples. The fact that already one week post inoculation these animals were RNA negative also shows that input virus did not interfere with the read-out. The mouse inoculated with Con1/K1846T virus also was readily infected and viremia was well detectable in all available serum samples.

To confirm that the viral genome in the mouse inoculated with the Con1/K1846T mutant had not reverted to wild type, the serum sample obtained at week 6 post inoculation was used for cloning of a genome fragment covering the NS4B coding region. Sequence analysis confirmed that the HCV genome in this mouse had retained this particular mutation (data not shown). In summary, these results convincingly demonstrate that Con1/wt and Con1/K1846T transfected Huh-7 cells release infectious HCV particles. Their production is blocked by various REMs, most notably those residing in the NS3 helicase. Thus, save for the single substitution in NS4B tested here, mutations that enhance RNA replication interfere with virus assembly.

## Discussion

Production of infectious HCV in cell culture so far is only possible with the genotype 2a isolate JFH-1 which replicates to very high levels without requiring REMs. In contrast, all genotype 1 isolates described until now replicate very poorly and need enhancing mutations. As shown in this report, at least in the context of Con1, but probably also for other genotype 1 isolates, with the exception of the K1846T substitution in NS4B REMs interfere with virus production. This is an important finding for two reasons: First, these results support earlier assumptions that REMs augment RNA-replication via different mechanisms [17]. While most of the mutations we analyzed more or less completely abolished virus production, the K1846T mutation in NS4B elevated RNA replication but interfered with virus formation only to a minor extent. These results clearly point to qualitative differences in the mode by which REMs modulate RNA replication and (in)-directly virus production.

Second, these data clarify why Huh-7 cells transfected with genomes containing REMs [25],[26] failed to produce virus particles. It is not due to unfavourable host cell conditions like the lack of assembly factors, but rather a consequence of these mutations especially those in NS3 that interfere with virus production, most likely particle assembly. These data therefore explain the attenuation of adapted Con1 genomes *in vivo* [27]. In fact, in an earlier study we had shown that a Con1 genome containing the three adaptive mutations of the Con1/NS3+S2197P construct (E1202G and T1280I in the helicase and S2197P in NS5A) was unable to establish an infection upon intrahepatic inoculation of a chimpanzee. A Con1 genome with only the NS5A mutation (S2197P) was attenuated and rapidly reverted to wild type. Taking the data from the present study into account, we can assume that these genomes replicated in RNA-inoculated hepatocytes of the chimpanzee, but due to impaired assembly progeny virus was not produced by Con1/NS3+S2197P and therefore the infection was abortive. Since the Con1/S2197P mutant still releases core protein (virus), albeit to very low levels, initial virus spread in the animal most likely was very limited until the mutant had reverted to wild type.

We note that REMs have also been described extensively for the genotype 1a isolate H77 [16]. These mutations reside primarily in the center of NS5A, but cooperative mutations have also been found in the helicase [42]. Although initial attempts to produce infectious H77 virus in cell culture failed with genomes carrying these mutations [42], a highly adapted genome containing 5 REMs has been described recently that replicates to levels comparable to JFH-1 [43],[44]. Most notably, cells transfected with this H77-S genome release infectious virus particles, but the amounts are very low. Moreover, the specific infectivity calculated as the ratio of HCV RNA molecules (genomes) per infectious unit was about 400-fold lower as compared to JFH-1 (5.4×10e4 vs. 1.4×10e2, respectively) [44]. The reason why H77-S transfected cells release such high amounts of HCV RNA is unclear. However, the low buoyant density of the RNA in density gradients and the presence of NS3 and NS5B in these fractions suggest that replication complexes possibly released from dying cells due to cytotoxicity of the efficiently replicating H77-S genome may in part account for these high RNA copy numbers in culture supernatants.

Owing to poor replication, infectivity assays of Con1/wt and Con1/K1846T viruses were extremely difficult. Although replication of the wild type genome could be stimulated with the kinase inhibitor H479, only a very low number of NS5A positive cells became detectable. In case of the Con1/K1846T genome, intrinsic replication efficiency of this genome was still too low for unambiguous detection of viral RNA or proteins. Inclusion of additional REMs either reduced RNA replication (in case of REMs residing in NS5A) [17] or blocked core release (in case of REMs residing in NS3). Furthermore, treatment of Con1/K1846T transfected cells with the kinase inhibitor H479 reduced rather than enhanced RNA replication, comparable to what has been described for REMs residing in NS5A [11]. Finally, attempts to adapt Con1/wt or Con1/K1846T genomes to continuous Huh-7 cell culture failed, because the genomes could not be maintained in passaged cells or culture supernatants, due to insufficient replication capacity (V.L. and R.B., unpublished).

Although the underlying mechanism interfering with virus assembly is unclear, our result argues for a cross-talk between structural and non-structural proteins during the assembly process. In fact, several TEMs have recently been described in the context of JFH-1 and various JFH-1-based infectious chimeras. These mutations reside in the region encoding core to NS2, but very often in the NS3 helicase domain and the RNA binding replicase factor NS5A [18]–[22], [45]–[47]. The mutations stimulate production of infectious virus particles without major effects on RNA replication arguing that the viral NS proteins modulate the efficiency of virus production [18]. In fact, we and others have recently shown that NS5A plays a very critical role in the assembly process, which occurs in close proximity of lipid droplets [6], [7], [48]–[50]. Core protein accumulates on the surface of these organelles and appears to recruit NS5A or the replicase complex to these sites to trigger virus assembly [48]. It was also found that alterations of NS5A phosphorylation, for which casein kinase I appears to play a major role [10],[38], have a strong impact on NS5A – core interaction and virus assembly [7],[8] and that most, if not all, REMs reduce NS5A hyperphosphorylation [16],[51],[52]. Finally, pharmacological inhibition of NS5A hyperphosphorylation enhances RNA replication as is the case with REMs [11]. The current model of HCV assembly that emerges from these observations assumes that via its domain 2 core protein efficiently localizes to lipid droplets [53] whereas NS5A is primarily a component of the replicase complex. We speculate that depending on its phosphorylation status, NS5A is recruited to lipid droplets to interact with the core protein in a way that the viral RNA genome is transferred to core, thus triggering virus assembly. In this respect REMs described here may interfere with the interaction between NS5A and core or recruitment of NS5A to lipid droplets or the RNA transfer from the replicase (helicase, NS5A) to the core protein thereby attenuating virus production. The fact that most REMs enhance RNA replication could therefore be due to retention of the viral RNA within the replication complex at the expense of RNA transfer to lipid droplets and/or RNA delivery to the core protein. In this context it is important to note that enhanced replication itself is not responsible for the interference with virus assembly since an adapted Con1 genome with an inactive NS5B polymerase still does not support virus production (data not shown) whereas the analogous replication deficient genome lacking REMs does (Fig. 1D). Moreover, JFH-1/wt supports assembly in spite of highly efficient RNA replication. Therefore, we hypothesize that REMs may arrest the viral RNA in a state that prevents the assembly process. The low-level release of virus from H77-S transfected cells may be due to an alternative assembly/release pathway that predominates under these experimental conditions. Clarification of these hypotheses requires more insights into the mechanisms of HCV particle assembly and release.

Although extensive tests with other HCV isolates have not been performed we hypothesize that non-adapted consensus genomes, at least those with proven *in vivo* infectivity, will also support production of infectious virus particles in transfected Huh-7 cells. However, owing to the very low replication levels of these genomes, demonstration of infection of cell cultures will be very difficult, even when stimulating replication e.g. by kinase inhibitors. As shown here, inoculation of xenografted mice with cell culture grown HCV particles is an alternative that is more robust and reliable. In fact, infection of uPA-SCID mice with supernatants of Con1/wt or Con1/K1846T transfected cells resulted in a well detectable viremia. In contrast, supernatants of Con1/NS3+K1846T transfected cells turned out to be non-infectious although these supernatants also contained viral RNA and low amounts of core protein. The nature of these RNA/core structures is not known but due to their low abundance they are not amenable to a biophysical characterization. They may correspond to lipid-containing replication complexes that were released from dying cells, similar to what we and others described earlier [25],[44].

For several positive strand RNA viruses it has been shown that RNA translation, replication and assembly are tightly coupled [54]–[57]. This coupling may act as a proof-reading mechanism to exclude from progeny particles those viral genomes that have a defect in either translation or RNA replication. As shown here a HCV genome that is unable to replicate (Con1/D318N; Fig. 1) still releases core protein to an amount comparable to the wild type. Although formal proof is missing that this core protein indeed corresponds to virus particles, our data suggest that HCV particle assembly may occur even in the absence of RNA replication.

In summary we demonstrate the production of infectious HCV particles in the Huh-7 cell line upon transfection with the genotype 1b isolate Con1. The interference of REMs with the assembly process provides an explanation why earlier attempts to produce infectious HCV in cell culture were of very limited success. Although this hurdle has in principle been overcome with the identification of the JFH-1 isolate, more replication and assembly competent HCV isolates are urgently needed to cover the full spectrum of genotypes, especially those that are poorly accessible to antiviral therapy. The observation that infectious HCV particles can be produced in Huh-7 cells by the genotype 1b isolate Con1 may provide a new starting point that likely can be extrapolated to other isolates with proven *in vivo* infectivity.

## Materials and Methods

### Cell culture

Huh-7 cell clones Huh7-Lunet [58] and Huh7.5 [42] that both are highly permissive for HCV RNA replication were used for electroporation and infection assays. Cells monolayers were grown in Dulbecco's Modified Eagle Medium ([DMEM] Life Technologies GmbH, Karlsruhe, Germany) supplemented with 2 mM L-glutamine, nonessential amino acids, 100 U of penicillin per ml, 100 µg of streptomycin per ml, and 10% fetal calf serum (complete DMEM). Cells were routinely subpassaged twice a week at a ratio of 1∶4 to 1∶10, depending on confluency. For infection experiments, Huh7.5 cells were seeded 24 h prior to infection into 12-well plates or on glass cover-slips contained in 24-well plates. Cell densities ranged from 2 to 5×10^4^ cells per well in case of a 12-well plate and 1 to 3×10^4^ cells per well in case of a 24-well plate.

### Plasmid construction

All full-length HCV Con1 constructs are based on the consensus clone of the HCV isolate Con1 [59] [AJ238799]. Generation of pFK-Con1/NS3+S2197P, pFK-Con1/NS5A and pFK-Con1/D318N (Con1/D318N) has been described recently [27]. Plasmid pFK-Con1/NS3+S2197P differs from pFK-Con1 by 5 nucleotide exchanges (A3946G, C4180T, C6842T, C6926T, and T6930C). Three of these mutations cause amino acid substitutions (E1202G, T1280I and S2197P), whereas the remaining changes are silent. Construct pFK-Con1/NS5A contains a single amino acid substitution (S2197P) and two silent nucleotide changes (C6842T and C6926T). The pFK-Con1/D318N plasmid encodes a replication-deficient variant of Con1 that carries a single amino acid substitution changing the GDD motif of the NS5B polymerase to D318N [15]. Constructs pFK-Con1/ΔE1-E2, pFK-Con1/A358ins and pFK-Con1/NK367AA were generated by PCR-based mutagenesis of pFK-Con1. PFK-Con1/ΔE1-E2 carries an in frame deletion encompassing amino acid residues 200 to 542 deleting most of the E1 and E2 coding region. Construct pFK-Con1/A358ins encodes a Con1 genome with an insertion of an alanine residue after amino acid 358 which is located in the transmembrane domain of E1. This mutation was shown to abrogate heterodimerization of E1 and E2 [31]. Variant pFK-Con1/NK367AA comprises two mutations in the transmembrane region of E1, replacing asparagines 367 and lysine 370 by alanine residues. Similar to A358ins, also this mutant was shown to abrogate heterodimerization of HCV glycoproteins [30]. Constructs pFK-Con1/K1846T and pFK-Con1/NS3+K1846T were generated by insertion of a *Sfi*I*-Sfi*I HCV genome fragment isolated from pFK-I341Luc/NS3-3′/K1846T and pFK-I341Luc/NS3-3′/ET [17]. Constructs pFK-Con1/NK367AA+K1846T and pFK-Con1/A358ins+K1846T were generated by insertion of a *Sfi*I*-Sfi*I HCV genome fragment isolated from pFK-Con1-K1846T. Generation of subgenomic replicons pFK-I389Luc/NS3-3′/wt and pFK-I389Luc/NS3-3′/GND has been already described [15]. Plasmids pFK-I389Luc/NS3-3′/K1846T and pFK-I389Luc/NS3-3′/NS3+K1846T were generated as described above by transfer of a *Sfi*I*-Sfi*I HCV genome fragment. All mutations were verified by DNA sequence analysis. The exact cloning strategies used to generate these constructs can be obtained upon request.

### *In vitro* transcription

PFK-based plasmids were restricted wit *Ase*I and *Sca*I, whereas puC-based plasmids were linearized with *Xba*I. Digested plasmids were extracted with phenol and chloroform, ethanol precipitated and dissolved in RNase-free water. *In vitro* transcription mixtures comprised 80 mM HEPES [pH7.5], 12 mM MgCl_2_, 2 mM spermidine, 40 mM dithiothreitol (DTT), each nucleotide triphosphate at a concentration of 3.125 mM, 1 U RNasin/µl of reaction volume, 0.1 µg restricted plasmid DNA/µl, and 0.6 U of T7 RNA polymerase/µl. Reactions were incubated for 2 h at 37°C, an additional 0.3 U T7 RNA polymerase/µl was added and the mixture as incubated another 2 h. Transcription was terminated by addition of 1.2 U of RNase free DNase (Promega) per µg of plasmid DNA and incubation for 30 min at 37°C. After extraction with acidic phenol and chloroform, RNA was precipitated with isopropanol and dissolved in RNase-free water. RNA concentration was determined by measuring absorbance at 260 nm, and the integrity of the transcripts was verified by denaturing formaldehyde agarose gel electrophoresis.

### Electroporation of Huh-7 cells and transient HCV replication assays

Single cell suspensions of Huh-7, Huh7-Lunet and Huh7.5 cells were prepared by trypsinization of monolayers. Detached cells were washed once with PBS and resuspended in cytomix [60] containing 2 mM ATP and 5 mM glutathione at a concentration of 1.5×10^7^ cells per ml in case of Huh7.5 cells or 1×10^7^ cells per ml in case of Huh-7 and Huh7-Lunet cells. Ten µg of *in vitro* transcript was mixed with 400 µl of the cell suspension, and electroporated at 960 µF and 270 V by using a Gene Pulser system (Bio-Rad, Munich, Germany) and a cuvette with a gap width of 0.4 cm (Bio-Rad). Depending on the amount of transfected cells required for the respective experiment either a single electroporation was performed, or cells from several electroporations were pooled and seeded into culture dishes.

### Preparation of total RNA and Northern blot analysis

Total cellular RNA for Northern blots was prepared by a single-step isolation method [61]. For RNA detection by quantitative PCR, the NucleoSpin RNAII kit (Macherey-Nagel, Düren, Germany) was employed and used according to the instructions of the manufacturer. For Northern blotting, total cellular RNA was denatured by treatment with 5.9% glyoxal in 50% dimethylsulfoxide and 10 mM sodium phosphate buffer [pH 7.0] at 50°C for 1 h. Subsequently, RNA was resolved by denaturing agarose gel electrophoresis and transferred to a positively charged nylon membrane (Hybond-N+; Amersham Pharmacia Biotech, Freiburg, Germany) with 50 mM NaOH using a vacuum manifold. After drying and crosslinking by UV irradiation, hybridization was performed according to standard protocols [62]. HCV-specific RNA was detected using a [^32^P]-labeled negative sense riboprobe complementary to NS5B and the 3′ UTR (nucleotides 8374–9440). HCV-specific bands were quantified by phosphimaging using a BAS 2500 scanner from Fuji.

### Quantitative detection of HCV core by ELISA and HCV RNA by PCR

HCV core protein expressed within cells or secreted into the culture medium was quantified using the commercially available Trak C Core ELISA (Ortho Clinical Diagnostics, Neckargemünd, Germany) according to the instructions of the manufacturer. When intracellular core expression was determined, cells were lysed in ice cold PBS supplemented with 1% Triton-X-100, 1 mM PMSF and 0.1 µg/ml Aprotinin. Lysates were cleared at 20,000×g for 10 min. and supernatants were measured at a dilution of 1∶50 (or higher) in PBS. Cell culture medium was filtered through 0.45 µm pore size filters and either directly used for ELISA or diluted with PBS prior to measurement.

### Quantitative detection of HCV RNA by qRT-PCR

Total RNA prepared from gradient fractions, infected cells or magnetic beads was eluted from NucleoSpin RNAII columns in a volume of 40 µl RNase-free water. Five microliters of the respective sample were used for quantitative RT-PCR analysis employing an ABI PRISM 7000 Sequence Detector (Taqman; Perkin-Elmer). Amplifications were conducted at least in duplicate with the One Step RT-PCR Kit (Qiagen, Hilden, Germany) using the following primers and 3′-phosphate-blocked, 6-carboxyfluorescine (6-FAM)- and tetrachloro-6-carboxyfluorescine (TAMRA)-labeled probes (TIB Molbiol, Berlin, Germany): HCV-Con1 Taqman probe, 5′-6FAM-TCC TGG AGG CTG CAC GAC ACT CAT-TAMRA-3′; HCV-Con1-S66, 5′-ACG CAG AAA GCG TCT AGC CAT-3′; and HCV-Con1-A165, 5′-TAC TCA CCG GTT CCG CAG A-3′; HCV-JFH1 Taqman probe,; HCV-JFH1-S147, 5′-TCT GCG GAA CCG GTG AGT A-3′; HCV-JFH1-A221, 5′-GGG CAT AGA GTG GGT TTA TCC A-3′. Reactions were carried out in three stages under the following conditions: stage 1, 60 min at 50°C (reverse transcription reaction); stage 2, 15 min at 95°C (heat inactivation of reverse transcriptase and activation of *Taq* polymerase); stage 3, 40 cycles, with 1 cycle consisting of 15 sec at 95°C and 1 min at 60°C. The total reaction volume was 15 µl and contained the following components: 2.66 µM 6-carboxy-X-rhodamine (Rox, passive reference), 4 mM MgCl_2_, 0.66 mM deoxynucleoside triphosphates, 0.266 µM probe, 1 µM (each) sense and antisense primer, and 0.6 µl of enzyme mix. The amounts of HCV RNA were calculated by comparison to serially diluted *in vitro* transcripts included in the qRT-PCR analysis.

### Ultracentrifugation

About 20 to 30 ml of filtered cell culture medium derived from Huh-7 cells 24 h or 72 h post transfection were concentrated via ultracentrifugation over a 40% or 60% (wt/vol) iodixanol (Optiprep; Invitrogen, Karlsruhe, Germany) density cushion (ρ = 1.215 g/ml, or 1.320 g/ml respectively), prepared in CSM (0.85% [wt/vol] NaCl, 10 mM Tricine-NaOH [pH 7.4]; ρ = 1,006 g/ml), in a SW28 rotor for 7 h at 100,000×g (RCF_avg_) at 4°C. Density cushion and interface were resuspended and used for infection or virus capture assays. Alternatively, resuspended material was transferred to the bottom of a fresh tube, and overlaid with a linear iodixanol gradient (60% to 0%) and spun for 18 h in a SW41 rotor at 110,000×g (RCF_avg_) at 4°C. Twelve fractions (1 ml each) were harvested from the top. The amount of HCV core protein in 100 µl of each fraction was determined by using Trak C ELISA. For quantifying HCV RNA, 100 µl of the respective gradient fraction were used for RNA preparation with the NucleoSpin RNAII kit (Macherey-Nagel, Düren, Germany). Five µl of the eluate (equivalent to 12.5% of the sample) were used for quantitative RT-PCR.

### Virus capture assay

For the production of lentiviral HCV pseudoparticles (HCVpp), 293T cells were transfected by using the calcium phosphate method essentially as described. Briefly, 2.5×10^6^ 293T cells were seeded in 10-cm diameter plates 1 day before transfection with 2.7 µg of phCMVΔCE1-E2(Con1), 8.1 µg of HIV-Gag-Pol expression construct [pCMV_R8.74 [63]], and 8.1 µg of the lentiviral vector pHR′-CMV-GFP [64] (where CMV is cytomegalovirus and GFP is green fluorescent protein). The medium was replaced 8 h after transfection. Supernatants containing the pseudo-particles were harvested 48 h later, cleared by passage through 0.45-µm-pore-size filters. Cleared HCVpp-containing culture fluids were used for immuno-capture assays. The equivalent of 25 µl of Dynabeads Protein A (Dynal, Invitrogen) slurry were washed according to the instructions of the manufacturer and coupled to 4 µg of human monoclonal antibody by continuous shaking at room temperature in a total volume of 25 µl sodium phosphate buffer [pH 8.1] for 40 min. Beads were washed 3 times with 0.5 ml sodium phosphate buffer and blocked 1 h at room temperature with 200 µl PBS, 2% bovine serum albumin. After 3 washes with PBS, beads were incubated with 100 µl filtered supernatant containing HCVpp for 1 h at 4°C by shaking, washed 5 times with PBS and resuspended in a final volume of 100 µl PBS. Bound p24 on the beads was measured by using the INNOTEST HIV Antigen mAb kit (Innogenetics) with minor modification of the protocol. In brief, beads were incubated with 100 µl conjugate buffer 1 to release bound p24 and centrifuged 1 min at 14,000 rpm. Supernatant was diluted 1∶50 into conjugate buffer 1 and further processed according to the standard protocol of the supplier. Human monoclonal antibodies employed for virus capture assays were described previously [65]. For capture of cell culture derived HCV Con1 particles, 5 µg of purified human monoclonal antibodies (CBH5, or the negative control RO4 antibody) coupled to protein A magnetic beads (Dynal) were incubated with filtered and concentrated cell culture medium for 1 h using an overhead rotor at 4°C. Beads were washed 5 times with 1 ml PBS, and RNA was extracted by using the NucleoSpin RNAII kit (Macherey-Nagel). Captured RNA was quantified by using TaqMan qRT-PCR.

### Characterization of virus particles by detergent and nuclease treatment

Virus particles contained in culture supernatant were concentrated as described above. For each capture assay 5 µg of CBH5 or RO4 antibody was covalently coupled to tosyl-activated Dynabeads (Invitrogen, Germany) according to the instructions of the manufacturer. Virus particles were captured as described above, beads were washed 5-times with 1ml of PBS supplemented with 1 mM CaCl_2_ and resuspended in 100 µl of the same buffer. Immune complexes were left untreated or incubated with 0.5% Triton X-100 for 5 min at RT in the presence or absence of 2.5 µl RNAsin (Promega, Mannhein, Germany). Thereafter, beads were incubated in the absence or presence of 2U S7 nuclease (Roche Mannheim, Germany) for 30 min at 37°C and the nuclease was inactivated by the addition of 2 mM EDTA. RNA was extracted by using the NucleoSpin RNAII kit (Macherey-Nagel, Düren, Germany) and RNA was quantified by using TaqMan qRT-PCR.

### Replication assays in the presence of casein kinase I inhibitor H479

Transient HCV RNA replication assays were performed as described previously above. Huh7.5 cells were electroporated with a subgenomic Con1/wt or Con1/K1846T or Con1/D318N or Con1/NS3+K1846T luciferase replicon and resuspended in 20 ml culture medium. Aliquots of 2 ml each were seeded per well of a 6-well plate and replication was determined by measuring luciferase activity 4, 24, and 48 h post-transfection. Four hours after electroporation, medium was removed and cells were treated with various concentrations of H479. Luciferase activities were normalized to the DMSO control value to determine the fold induction of replication. Statistical analyses were conducted using PRISM4 (GraphPad Software Inc.) and two-way ANOVA test.

### Production of concentrated culture supernatants, cell culture infectivity assay and immunofluorescence

Supernatants from 10 electroporations of Huh7.5 cells with each 10 µg Con1/wt or Con1/K1846T or Con1/NS3+K1846T were harvested 12 and 24 h post electroporation and pools were filtered through 0.45 µm-pore-size filters. Filtrates were loaded onto a cushion composed of 4 ml 10% Optiprep-PBS and 3 ml 28% Optiprep diluted in serum-free-medium. Samples were centrifuged for 4 h at 100,000×g in a SW28 rotor (Beckman) at 4°C. Virus concentrated at the interface of the cushion was recovered and concentrated by centrifugation using a Centricon Plus-70 centrifugal filter device (100K NMWL; Millipore, Germany) according to the instructions of the manufacturer. Concentrated virus was resuspended in 1 ml culture medium. Based on core-ELISA measurements we calculated that about 50% of core protein was recovered in this final preparation and that core protein was concentrated 200-fold. Huh7.5 cells seeded on glass cover slips were pretreated with or without 25 nM of Concanamycin A for 1 h at 37°C; thereafter cells were infected with 300 µl of the concentrate in the presence or absence of 25 nM Concanamycin A (Sigma). Four hours later cells were washed with fresh medium and incubated in complete medium with or without H479 (10 µM). After 72 h, cells were fixed with icecold methanol. Immunolabelling of NS5A was performed with a monoclonal antibody specific for NS5A (Virostat, Portland, USA) at a dilution of 1∶50 in PBS supplemented with 5% normal goat serum. NS3 was detected with a rabbit polyclonal antiserum obtained by immunization with a recombinant NS3 fragment of JFH-1 (amino acid residues 293 to 631). Bound primary antibodies were detected by using a goat antibody conjugated to AlexaFluor488 (Invitrogen, Germany) at a dilution of 1∶1,000 in PBS/5% normal goat serum. Nuclear DNA was counterstained with DAPI (Molecular Probes, Eugene, OR). Images were acquired with an inverted fluorescence microscope (Leica, Germany).

### Infection of uPA+/+-SCID mice

The mouse study was conducted at the Ghent University Hospital, with protocols approved by the Ethical Committee and Animal Ethics Committee of the Ghent University Faculty of Medicine. Transgenic SCID mice overexpressing the uPA gene under the control of an albumin promoter (uPA^+/+^-SCID) were xenografted with primary human hepatocytes as described elsewhere [66]. Chimeric mice were inoculated by intraperitoneal injection of 100 µl of purified and concentrated culture supernatant (prepared as described above). Inocula contained 2×10^8^ IU HCV RNA for each preparation and 540 pg core protein for Con1/wt, 200 pg for Con1/K1846T and 23 pg core protein for Con1/NS3+K1846T. EDTA plasma samples were collected at weekly intervals after inoculation and infection was monitored by a commercial qRT-PCR kit (Roche COBAS AmpliPrep/TaqMan48 assay, Roche Diagnostics). Due to dilution of the samples, the detection limit of the test was 750 IU/ml.

### Preparation of total RNA from mouse serum, amplification of HCV RNA by RT-PCR and sequence analysis of cloned amplicons

HCV RNA was isolated from 110 µl of serum taken from mouse K831 six weeks post inoculation with Con1/K1846T. Viral RNA was isolated by using the Nucleo Spin RNA Virus Kit (Macherey-Nagel, Germany) as recommended by the manufacturer. RT-PCR was performed with the Expand-RT system (Roche, Germany) according to the instructions of the manufacturer using primer A-9413 (5′-CAG GAT GGC CTA TTG GCC TGG AG-3′) for cDNA synthesis. First amplification was done by using the Expand Long Template PCR Kit (Roche) and primers S-4542 (5′-GAT GAG CTC GCC GCG AAG CTG TCC-3′) and A-6156 (5′-CGC TCT CAG GCA CAT AGT GCG TGG-3′). Because of low RNA levels nested PCR was required, for which we used primers S-4542 and A-6103 (5′-GCT ATC AGC CGG TTC ATC CAC TGC-3′). Amplified fragment was inserted into pFK-I389Luc-EI/NS3-3′/JFH1-dg after restriction with *Nsi*I and *Hpa*I. Sequence analysis was performed using primer A-8242 (5′- CGT TGG GCA GGG GAG TAC TGG AAG -3′).
